# Enhanced Photovoltaic Properties in Sb_2_S_3_ Planar Heterojunction Solar Cell with a Fast Selenylation Approach

**DOI:** 10.1186/s11671-018-2651-x

**Published:** 2018-09-06

**Authors:** Kun Wang, Jiang Cheng, Xin Yang, Rong Hu, Lijuan Fu, Jiang Huang, Junsheng Yu, Lu Li

**Affiliations:** 1 State Key Laboratory of Electronic Thin Films and Integrated Devices, School of Optoelectronic Science and Engineering, University of Electronic Science and Technology of China (UESTC), Chengdu, 610054 People’s Republic of China; 2 Co-Innovation Center for Micro/Nano Optoelectronic Materials and Devices, Research Institute for New Materials and Technology, Chongqing University of Arts and Sciences, Chongqing, 402160 People’s Republic of China

**Keywords:** Antimony chalcogenide, Sb_2_S_3_ solar cell, Selenylation, Energy level, Thermal evaporation

## Abstract

Poor thermostability of Sb_2_S_3_ in vacuum hinders the possibility of achieving high-quality crystalline films. In order to enhance the photovoltaic properties of Sb_2_S_3_ planar heterojunction solar cells, a selenylation-based post-treatment approach has been employed. Selenylation performed over 15 min on the Sb_2_S_3_ film resulted in an enhancement in the conversion efficiency from ~ 0.01 to 2.20%. Effect of the selenylation on the evolution of morphology, crystal structure, composition distributions, and photovoltaic behavior has been investigated. The variation in the energy levels of Sb_2_S_3_/CdS junction has been also been discussed. Results show that selenylation not only enhanced the crystallinity of Sb_2_S_3_ film but also provided a suitable energy level which facilitated charge transport from absorber to the buffer layer.

## Background

Inorganic thin film solar cells have received much attention owing to the advantages of being low cost and lightweight as compared to their silicon counterparts [1, 2]. They are chemically and physically stable in air in contrast to organic and organic–inorganic hybrid perovskite solar cells and have achieved very long operating lifetimes under practical settings [3–5]. Among them, copper indium gallium selenide (CIGS)-based and cadmium telluride (CdTe)-based solar cells are promising and have realized a conversion efficiency of 21.7% and 19.6% [6, 7], respectively. In recent years, another candidate material Cu_2_ZnSnS_*x*_Se_4 − *x*_ (CZTSSe) has been investigated as it is earth-abundant and has an environment-friendly composition [8, 9]. Although an impressive conversion efficiency of 12.6% has been achieved by a hydrazine-based solution process, this compound encountered complexities in terms of phase and defect control [10]. Additionally, the toxicity of hydrazine has seriously limited its further application [11–13]. Recently, binary antimony sulfide (Sb_2_S_3_) has gained importance as a thin film solar cell application, owing to its earth abundance, low cost, and the relatively low toxic composition of Sb and S elements [14, 15].

Sb_2_S_3_ exhibits a tunable energy bandgap (1.1–1.7 eV) when S elements are partly or completely replaced by Se, suggesting good designability of Sb_2_S_3_ for photovoltaic device preparation [16–18]. To date, various approaches have been reported to fabricate Sb_2_S_3_ absorber layers. Sb_2_S_3_-sensitized inorganic–organic heterojunction solar cells exhibit a high solar energy harvesting ability and have demonstrated conversion efficiency of 7.5% [19]. However, the device fabrication is complicated, and the lifetime of organic hole transporting materials is low. In comparison, planar heterojunction solar cells are advantageous in terms of simplified absorber preparation as well as device fabrication. Both physical vapor-deposited and solution-processed Sb_2_S_3_ films have been previously applied in planar heterojunction solar cell fabrication. All-inorganic Sb_2_S_3_ planar heterojunction devices with a simple structure of FTO/n-type buffer layer/Sb_2_S_3_/electrode have achieved a power conversion efficiency (PCE) of 1.27–4.17% [20–24]. Vacuum-based film deposition methods like magnetron sputtering are convenient to operate and provide accurate thickness control, reproducibility, and smooth surface building. Due to these advantages, they have been widely applied in industrial manufacturing of CIGS and CdTe solar cells. Sb_2_S_3_ has a low melting point (550 °C) and high vapor pressure, favoring thermal evaporation instead of magnetron sputtering. However, Sb_2_S_3_ exhibits a poor thermostability in vacuum resulting in significant deviations in the composition [25], and the tendency for surface oxidation. Currently, component preserving rapid thermal evaporation (RTP) has been employed in the fabrication of all-inorganic Sb_2_S_3_ solar cells having achieved a maximum PCE of 4.17% [23]. Compared with the rapid thermal evaporation technique, regular thermal evaporation has some advantages in terms of providing accurate thickness and variable morphology control. Also, the substrate rotation is easier to realize and beneficial for uniform preparation of large-area thin film specimen. Since the distance between substrate and source is greater, the required evaporation power is lower than rapid evaporation. This ensures that the source has less thermal effect on the substrate during evaporation process. It consumes less material and has better prospect in flexible solar cell fabrication. However, this approach has some limitations that need to be addressed. To avoid decomposition and surface oxidation, Sb_2_S_3_ films can only be prepared at a low substrate temperature (~ 200 °C) by regular thermal evaporation. However, the low substrate temperature resulted in poor crystallinity of the films, which was not suitable for the preparation of efficient photovoltaic devices.

Post-treatments including vacuum annealing and selenization for thermally evaporated Sb_2_S_3_ have been considered. In this study, rapid thermal processing technique has been utilized for the thermal treatment. Photovoltaic properties of Sb_2_S_3_ planar heterojunction device showed a considerable enhancement after several minutes of selenization. Processing conditions and the effect on the crystal structure and surface morphology were investigated. The formation of gradient composition, evolution of energy levels, and electronic behavior in device has also been discussed in detail. After the optimization of the technique, the PCE of planar photovoltaic devices showed a satisfactory improvement from ~ 0.01 to 2.20%.

## Methods/Experimental

A simple superstrate device structure (FTO (SnO_2_:F)/CdS/ Sb_2_S_3_/Au) was applied for the Sb_2_S_3_ films solar cells. FTO-coated glass (Pilkington, Toledo, USA) with a sheet resistance of 7 Ω/□ was used as the bottom electrode to collect electrons. A CdS buffer layer with a thickness of 90 nm was deposited on the FTO glass using the chemical bath deposition (CBD) method [26]. Sb_2_S_3_ films were thermally evaporated using Sb_2_S_3_ powder (aladin, 99.9%, Aladdin) at less than 5 × 10^−4^ Pa when the substrate temperature was kept at 175 °C, and then cooled down to room temperature naturally. The sample was then transferred into a two-zone tube RTP furnace at 10^3^ Pa in protective N_2_ atmosphere. Excess selenium powder was placed in a quartz boat in the low temperature zone (350 °C) whereas the sample was placed in the high temperature zone (400 °C). Subsequently, 60-nm Au electrode was deposited on the surface of absorber layer using DC magnetron sputtering.

Current density–voltage (*J*-*V*) characteristics were measured using a Keithley 2400 unit under an AM 1.5 (100 W/cm^2^) xenon lamp illumination (Newport 94043A). External quantum efficiency (EQE) of Sb_2_S_3_(Se) thin films were obtained using an integrated measurements system (Beijing SOFN 7-SCSpecIII). The crystal structure and composition were characterized by X-ray diffraction (XRD, Bruker D8). Optical property was characterized by ultraviolet–visible near infrared transmission spectroscopy (UV-Vis, Agilent Cary5000). Ultraviolet photoelectron spectroscopy (UPS, Thermo ESCALAB 250Xi) was used to determine the energy levels of the important photovoltaic layers. Surface morphology of Sb_2_S_3_(Se) films growth on CdS was characterized by scanning electron microscopy (SEM, FEI Helios Nanolab 600i). Carrier transporting behavior was investigated by electrochemical impedance spectrum (EIS) under a suitable open-circuit voltage.

## Results and Discussion

**Fig. 1:**
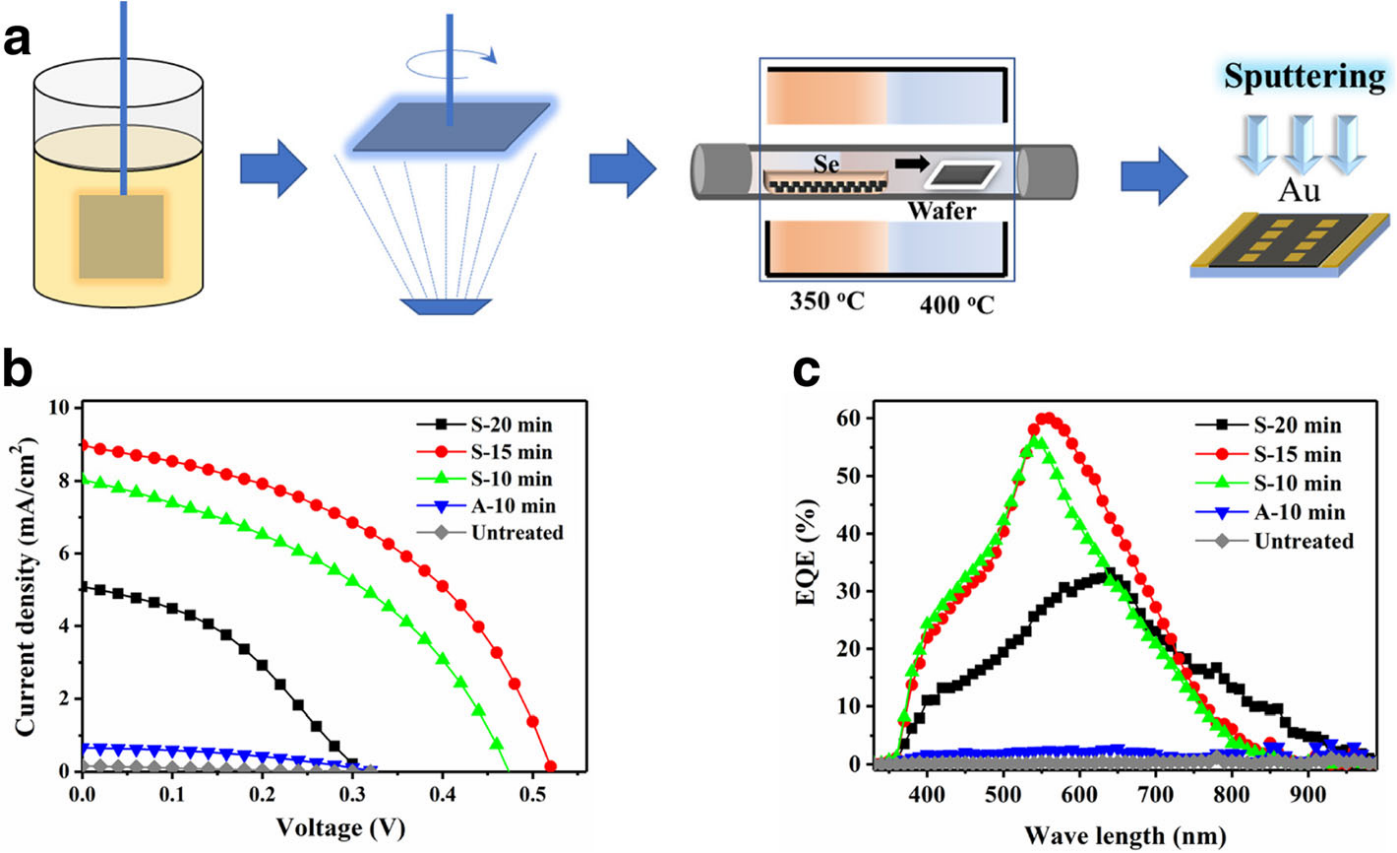
Device fabrication and photovoltaic performance. **a** Schematic diagram of the fabrication of selenized Sb_2_S_3_ photovoltaic devices. **b** *J*-*V* characteristics under illumination. **c** EQE of Sb_2_S_3_ photovoltaic devices under different treatment conditions

The schematic of the entire device fabrication procedure is shown in Fig. 1a. Each sample comprised of eight cells with a 4-mm^2^ active area that was tested under the same condition. Typical *J*-*V* characters of untreated, vacuum-annealed (A) and selenized (S) Sb_2_S_3_ are shown in Fig. 1b and their corresponding performance is summarized in Table 1. Untreated Sb_2_S_3_ device showed a low average PCE < 0.01% with an open-circuit voltage (*V*_OC_) of 0.31 V and a short current density (*J*_SC_) of 0.14 mA/cm^2^_._ After a 10-min vacuum annealing step, a minor improvement was obtained with *J*_SC_ = 0.66 mA/cm^2^ and a PCE = 0.08%. In contrast, selenized devices showed a significant enhancement in both *V*_OC_ and *J*_SC_ as compared to the untreated device with *J*_SC_ = 7.80 mA/cm^2^ and PCE = 1.57%. The best performance of device with a maximum PCE = 2.20% and a *J*_SC_ = 9.04 mA/cm^2^ was obtained when the selenization time was increased to 15 min. Increasing the selenization time beyond 15 min did not bring a further improvement of performance. For a selenization time of 20 min, the average PCE decreased to 0.61% due to the degeneration of both *V*_OC_ and *J*_SC_. Further extension of selenization time beyond 30 min resulted in a poor yield. EQE of the devices with the selenization effect is shown in Fig. 1c, where it is evident that the spectral response of the treated devices is significantly higher as compared to the untreated devices. This trend is well consistent with *J*-*V* characteristics of devices. Devices selenized for 15 min have the highest EQE, indicating a good spectral response in visible range. EQE peaks also show gradual red shift, and the spectral response ranges become wider with increasing selenization time. For the devices selenized for 20 min, a much wider EQE range from 350 to 400 nm is observed, which can be attributed to the composition change near p-n junction in the Se atmosphere annealing.

**Table 1:** The performances of Sb_2_S_3_ solar cells fabricated with different treatment conditions

Treatment	*V*_OC_ (V)	*J*_SC_ (mA/cm^2^)	FF (%)	PCE (%)	${\overline{\mathrm{R}}}_S$ (Ω cm^2^)	${\overline{\mathrm{R}}}_{sh}$ (Ω cm^2^)
Untreated	0.31 ± 0.02	0.14 ± 0.05	24.08 ± 2.55	≤ 0.01	1893	1721
Annealed 10 min	0.33 ± 0.02	0.66 ± 0.19	39.01 ± 1.73	0.08 ± 0.02	245	1978
Selenized 10 min	0.47 ± 0.03	7.80 ± 0.33	42.45 ± 0.86	1.57 ± 0.06	20	186
Selenized 15 min	0.52 ± 0.02	8.78 ± 0.26	46.35 ± 1.13	2.13 ± 0.07	15	188
Selenized 20 min	0.30 ± 0.07	5.13 ± 0.49	38.72 ± 3.28	0.61 ± 0.26	41	224

**Fig. 2:**
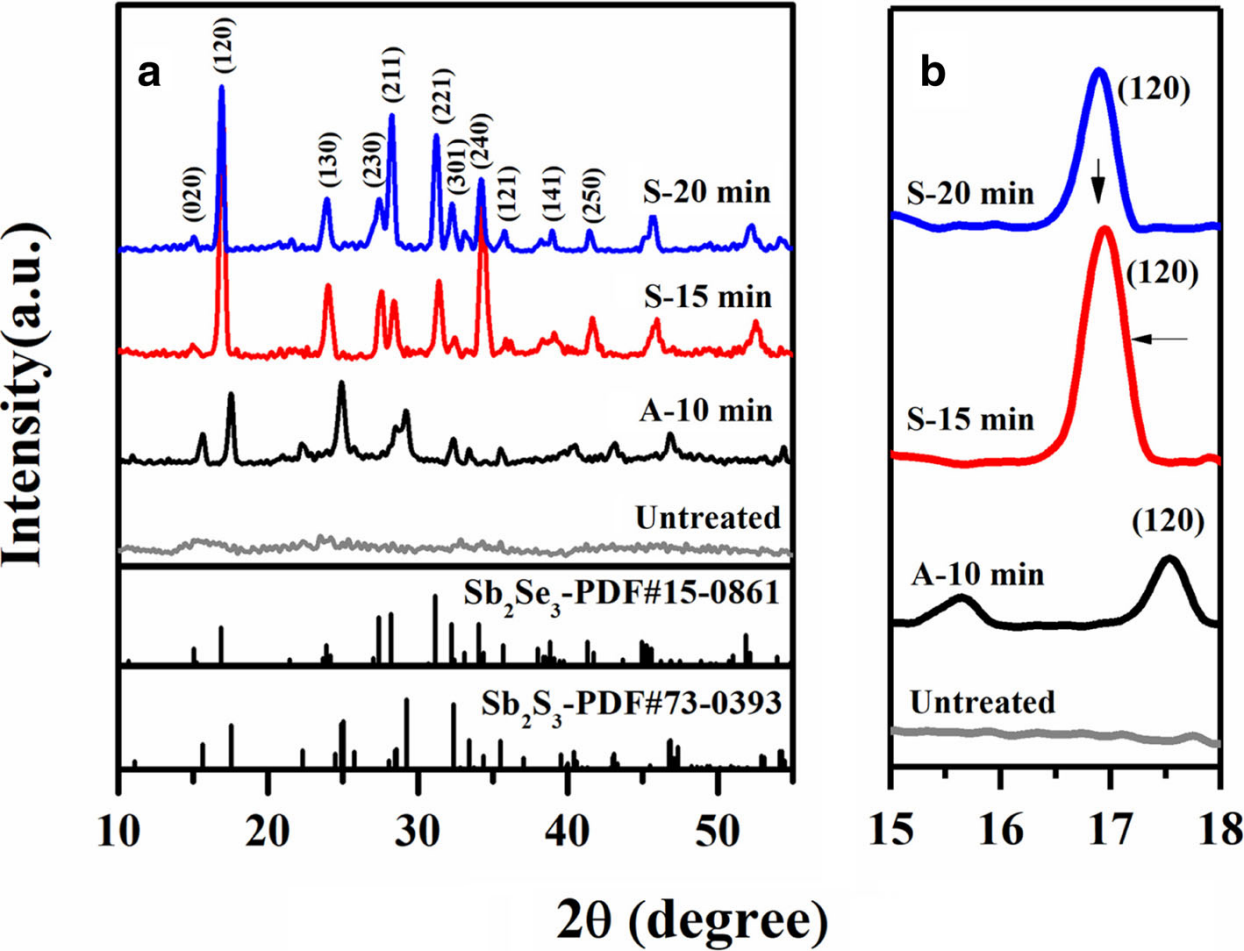
Crystal structure characterization of Sb_2_S_3_(Se) films. **a** XRD patterns of the Sb_2_S_3_ films under various treatment conditions. **b** Enlarged (120) XRD peaks of the same films as in **a**

XRD analysis was used to determine the overall crystal structure of the films under annealing and selenization treatment. As shown in Fig. 2a, untreated Sb_2_S_3_ films showed weak and indistinct XRD peaks indicative of low crystallinity, which explains the poor PCE with the low *J*_SC_. Vacuum-annealed and selenized films showed better crystallinity with distinguishable diffraction peaks, which was approximately matched to orthorhombic Sb_2_S_3_ (JCPDS NO. 15-0861). All diffraction peaks of selenized films gradually shifted to smaller 2*θ* angle as the selenization time increased. From the magnified (120) diffraction peaks shown in Fig. 2b, the 2*θ* value of Sb_2_S_3_ was found to be 17.50°, which shifted to 16.95° after a 15-min selenization time. The diffraction patterns tend to match the standard Sb_2_Se_3_ PDF card (JCPDS NO. 73-0393). Hence, it can be concluded that there was an increase in the lattice constant after selenization, where smaller S atoms (1.84 Å) were partly replaced by larger Se atoms (1.98 Å).

**Fig. 3:**
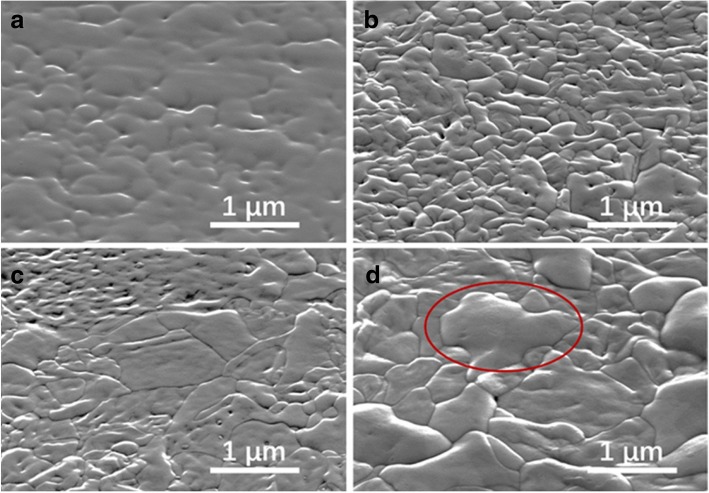
Top-view SEM images of Sb_2_S_3_ films under various treatment conditions. **a** Untreated. **b** Vacuum annealed. **c** Selenized for 15 min. **d** Selenized for 20 min

Further selenization (20 min) was observed to bring about a minor shift in the (120) XRD from 16.95° to 16.90°. We deduced that the replacement reaction rate decreased rapidly in the selenization process. The untreated film exhibited an amorphous textured surface, and the small-sized grains on the surface became more prominent when the film was vacuum annealed to 400 °C. A 15-min selenization treatment led to the formation of micron-sized large grains, indicating selenization can effectively promote the growth of grains which is consistent with XRD results. The compact surface hindered the replacement diffusion of Se, thus decreasing the reaction rate rapidly. The film selenized for 20 min showed large grains with distinct edges in the SEM image in Fig. 3. However, some bulges (red ellipse in Fig. 3d) can be observed on the surface that were responsible for the poor contact between absorber and the buffer layer. Accordingly, the 20-min selenized device exhibits a poor *J*_SC_ with a high series resistance (*R*_s_) as shown in Table 1. Further, prolonging the selenization time resulted in bulges evolving into cracks and short-circuiting the device.

**Fig. 4:**
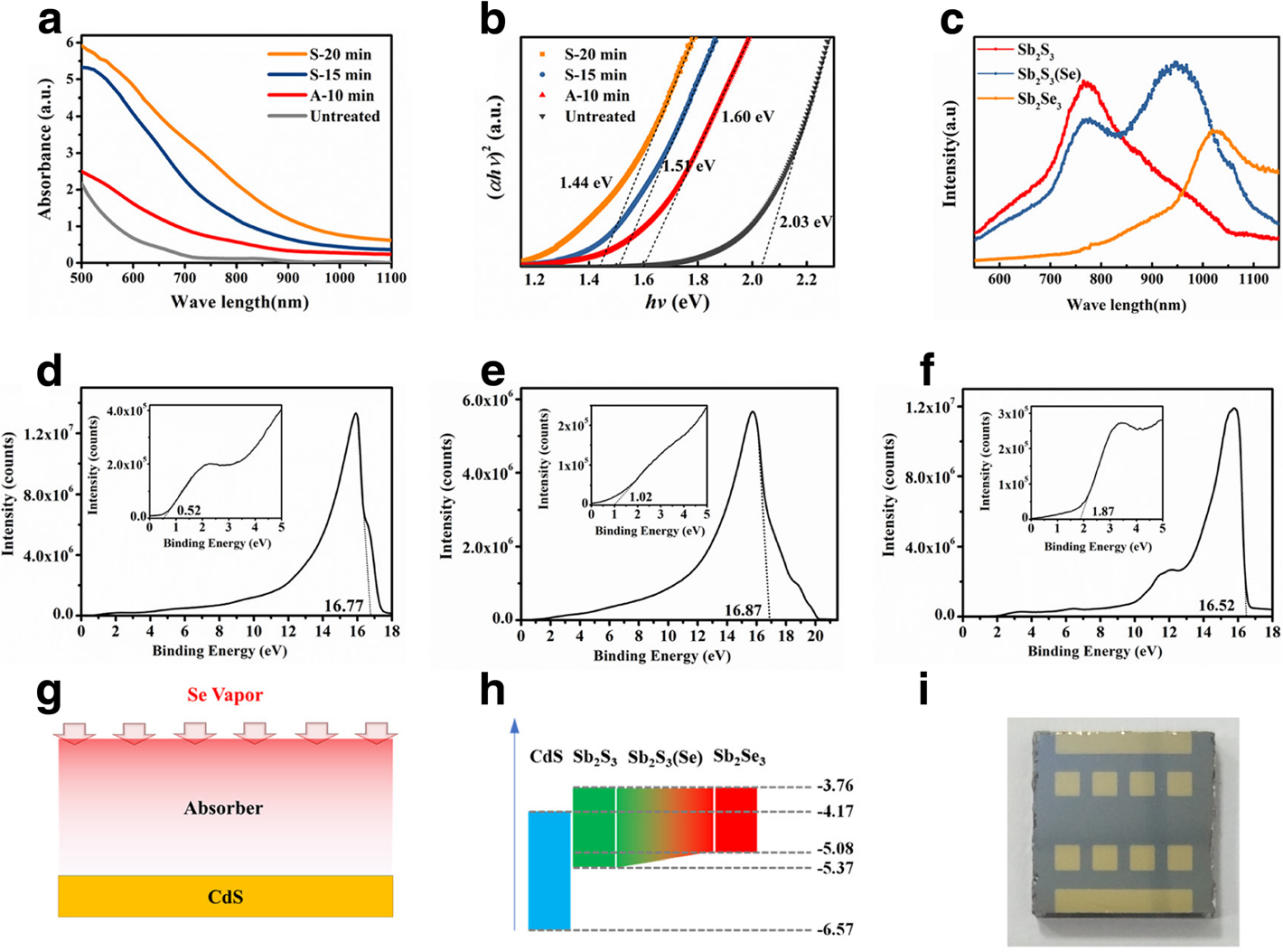
Energy level analysis of Sb_2_S_3_(Se) solar cells. **a** Ultraviolet–visible near infrared transmission spectroscopy (**b**) variation of (*αhv*)^2^ as a function of the photon energy (*hv*) of Sb_2_S_3_ films under different treatment conditions. **c** PL spectrum of vacuum prepared Sb_2_S_3_, Sb_2_S_3_(Se), and Sb_2_Se_3_ films. UPS spectra of **d** Sb_2_Se_3_, **e** Sb_2_S_3_, and **f** CdS. **g** Model of composition distribution and **h** energy levels along vertical depth of selenized Sb_2_S_3_ film. **i** An image of selenized Sb_2_S_3_ devices sample

To investigate the treatment effect on Sb_2_S_3_ energy level, the absorption spectrum from 500 to 1100 nm was measured by UV-Vis spectroscopy. As shown in Fig. 4a, both vacuum-annealed and selenized films show improved optical absorption. The absorbance profile showed a gradually increasing and long wave absorption edge with a continuous red shift as the selenization time increased. This indicates that the selenization process decreases the energy gaps. Since Sb_2_S_3_ is a direct band gap semiconductor, the band gap (*E*_g_) can be calculated by the Tauc formula [27]:

1 $$ \alpha =\left(A/ h\nu \right)\times {\left( h\nu -{E}_g\right)}^{1/2} $$

where *A* is a constant, *h* is the Planck’s constant, and *ν* is the frequency of the incident photon. *E*_g_ was determined from the linear fit of (*αhv*)^2^ versus (*hv*), as shown in Fig. 4b. *E*_g_ of untreated Sb_2_S_3_ film is 2.03 eV which decreased to 1.60 eV after annealing. The *E*_g_ gradually decrease to 1.44 eV as the selenization time increased to 20 min. To verify this, photoluminescence (PL) spectrum of films excited by a 325-nm laser was carried out. As shown in Fig. 4c, the PL peak of Sb_2_S_3_ was observed at 772 nm (1.61 eV) with a very small Stokes shift (0.01 eV), which is consistent with the optical band gap. Interestingly, PL spectrum of Sb_2_S_3_ selenized for 15 min splits into two peaks, one of which is located at 765 nm (1.62 eV) and the other located at 948 nm (1.31 eV). The 765-nm PL peak is very close to Sb_2_S_3_ peak (772 nm), implying that the composition deep within the Sb_2_S_3_ film remains virtually unchanged after 15 min of selenization. To study the energy level as well as carrier transport properties of photovoltaic devices, UPS analysis of Sb_2_Se_3_, Sb_2_S_3_, and CdS was carried out as shown in Fig. 4c–f. The energy level conduction band minimum (*E*_C_) and valence band maximum (*E*_V_) was determined as listed in Table 2. In accordance with the XRD and PL results, a replacement diffusion model is proposed, in which a substantial proportion of S in the surface is replaced by Se while the composition near p-n junction remains as Sb_2_S_3_ (Fig. 4g). The energy levels can be represented as shown in Fig. 4h. A group of selenized Sb_2_S_3_ devices is shown in Fig. 4i. Compared with vacuum-annealed Sb_2_S_3_/CdS device, selenized device had a satisfactory built-in electrical field at the p-n junction due to the favorable *E*_g_ of Sb_2_S_3_ (1.61 eV) which provided a higher *V*_OC_ than Sb_2_Se_3_ (*E*_g_ = 1.2 eV) [28, 29]. Due to the gradient distribution of composition, selenized Sb_2_S_3_ showed a continuous *E*_v_ varying from − 5.37 to − 5.08 eV and a lower barrier for photogenerated positive carrier transport from p-n junction vicinity to the anode. Accordingly, a considerable improvement of *J*_SC_ was realized, resulting in a higher PCE.

**Table 2:** Energy band information calculated from UPS spectra

Materials	*E*_g_ (eV)	VBM (eV)	Ws (eV)	*E*_c_ (eV)	*E*_v_ (eV)
CdS	2.40	1.87	4.70	− 4.17	− 6.57
Sb_2_Se_3_	1.21	0.52	4.45	− 3.76	− 5.08
Sb_2_S_3_	1.61	1.02	4.35	− 3.76	− 5.37

**Fig. 5:**
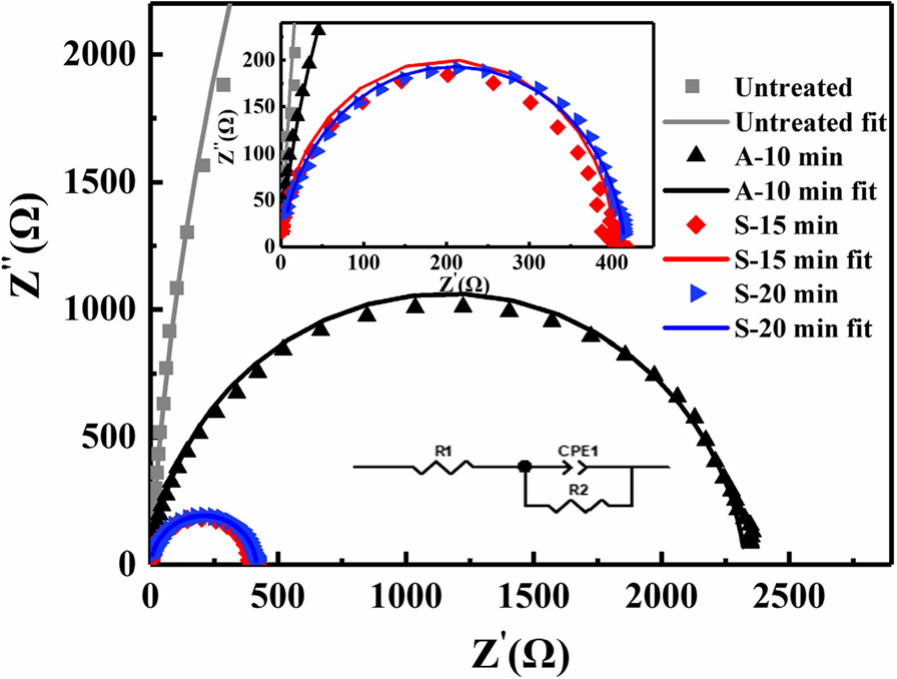
Impedance spectra of Sb_2_S_3_ under various treatment conditions measured in the dark, inset showing the overall narrowing diagram and equivalent circuit

To validate the selenization effect on the electronic behavior in the photovoltaic device, electrochemical impedance measurements were carried out, as shown in Fig. 5 along with simulations. For the planar heterojunction device, testing curves obey a semi-circular profile. Resistance-constant phase element (R-CPE) series equivalent electrical circuit model was applied to simulate the test results [30–32]. Series resistance *R*_1_ represents all factors that affect photogenerated carrier transport to electrodes, mainly the carrier transport resistance of photovoltaic films and electrodes. In this study, interface effect on resistance between Au and absorber is negligible due to the ohmic contact, and the major differences arise due to the absorber being treated under different conditions. Accordingly, *R*_1_ value is only related to positive carrier transport from absorber to Au electrode. The shunt pair *R*_2_ and CPE1 are associated with the interface between absorber and CdS buffer layer. CPE can be defined by the capacitance (CPE-T) and a non-homogeneity constant (CPE-P). All computed parameters from the fitted plot are listed in Table 3. There is no noticeable difference of CPE-T between the tested samples, and the value is in the 0.94–0.96 range, indicating all the devices could be treated as ideal capacitors with ideal interfaces. The magnitude of *R*_1_ was observed to be largely dependent on the treatment conditions. For the untreated device, *R*_1_ = 519.8 × 10^−3^ Ω cm^2^, which decreased to 10.0 × 10^−3^ Ω cm^2^ after a vacuum annealing process. For the device selenized for 15 min, the minimum *R*_1_ of 0.4 × 10^−3^ Ω cm^2^ was determined. The decreasing of *R*_1_ indicates vacuum annealing or selenization facilitated charge transport from absorber to the buffer layer. For the device selenized for 15 min, *R*_1_ increased to 815.5 × 10^−3^ Ω cm^2^ with a lower CPE-T of 0.84 10^−7^ F cm^−2^. The degradation was likely caused by poor interface contact between bulged absorber and the CdS buffer layer.

**Table 3:** Calculation result of equivalent circuit parameters from the fitted impedance spectrum

Absorber	*R*_1_ (10^−3^ Ω cm^2^)	CPE1-T (10^−7^ F cm^−2^)	CPE1-P	*R*_2_ (Ω cm^2^)
Untreated	519.8	1.38	0.96	26,666
Annealed 10 min	10.0	1.86	0.94	2358
Selenized 15 min	0.4	1.74	0.95	405.3
Selenized 20 min	815.5	0.84	0.94	426.3

## Conclusions

The selenization approach enhanced the crystallinity of Sb_2_S_3_ film and resulted in an improvement in the photovoltaic performance of device. Selenized Sb_2_S_3_ films exhibit a gradient composition distribution due to the partial replacement of S atoms by Se atoms in near surface while the bulk composition remains virtually unchanged. Thus, selenized film showed a consecutive Sb_2_S_3_/Sb_2_S_3_(Se)/Sb_2_Se_3_ structure that decreased the potential barrier for photogenerated positive carrier transport from the p-n junction vicinity to the anode. The optimal selenization conditions involves maintaining Se at 350 °C and Sb_2_S_3_ at 400 °C, with a selenization time of approximately 15 min. Excessive selenization time tends to introduce some bulges leading to a poor interface contact between absorber and CdS buffer layer, resulting in a poor performance and yield.

## Abbreviations

A: Annealed
CBD: Chemical bath deposition
CIGS: Copper indium gallium selenide
CZTSSe: Cu_2_ZnSnS_*x*_Se_4 − *x*_
EIS: Electrochemical impedance spectrum
EQE: External quantum efficiency
FTO: (SnO_2_:F)
*J*_SC_: Short current density
*J*-*V*: Current density–voltage
PCE: Power conversion efficiency
PL: Photoluminescence
R-CPE: Resistance-constant phase element
RTP: Rapid thermal processing
S: Selenized
SEM: Scanning electron microscopy
UPS: Ultraviolet photoelectron spectroscopy
UV-Vis: Ultraviolet–visible near infrared transmission spectroscopy
*V*_OC_: Open-circuit voltage
XRD: X-ray diffraction
